# A Rare Case of Small Bowel Injury From an Industrial High-Pressure Water Jet

**DOI:** 10.7759/cureus.102870

**Published:** 2026-02-03

**Authors:** William Swee Keong Khoo, Reizal Mohd Rosli, Adrian Sebastian

**Affiliations:** 1 Department of General Surgery, Townsville Hospital, Townsville, AUS

**Keywords:** abdominal trauma, general surgery, general trauma surgery, industrial high-pressure fluid injection injuries, small bowel injury, small-bowel injury

## Abstract

Industrial high-pressure fluid injection injuries are rare causes of abdominal trauma. The spectrum of injury ranges from superficial skin abrasions to severe intra-abdominal injuries, including hollow viscus perforation and solid organ damage, which may be life-threatening if not promptly recognised and managed. Furthermore, this mechanism of injury does not clearly conform to traditional classifications of either penetrating or blunt abdominal trauma; rather, it represents a hybrid mechanism often known as a hydroblast injury. This poses challenges for clinicians in applying established management algorithms. This case highlights the importance of maintaining a high index of suspicion and performing serial abdominal examinations, which facilitated early surgical intervention and led to the identification of a small bowel injury caused by an industrial high-pressure water jet.

## Introduction

Industrial high-pressure fluid injection injuries (IHPFII) are rare forms of trauma reported in the literature and most frequently involve the upper limbs, followed by the lower limbs, chest, and abdomen. In the setting of abdominal trauma, these injuries may present with a wide clinical spectrum, ranging from abdominal wall contusions or haematomas to solid organ injury with haemoperitoneum or hollow viscus perforation resulting in peritonitis [1]. This report describes a rare case of small bowel injury resulting from an industrial high-pressure water jet.

## Case presentation

A 66-year-old man was injured by an industrial high-pressure water jet while at work that had lost control and struck his lower abdomen and left flank. The estimated pressure of the water jet was approximately 4,000 pounds per square inch (psi). Upon arrival to the emergency department four hours from the onset of injury, the patient was systemically well, and vital signs were within normal limits.

**Figure 1:**
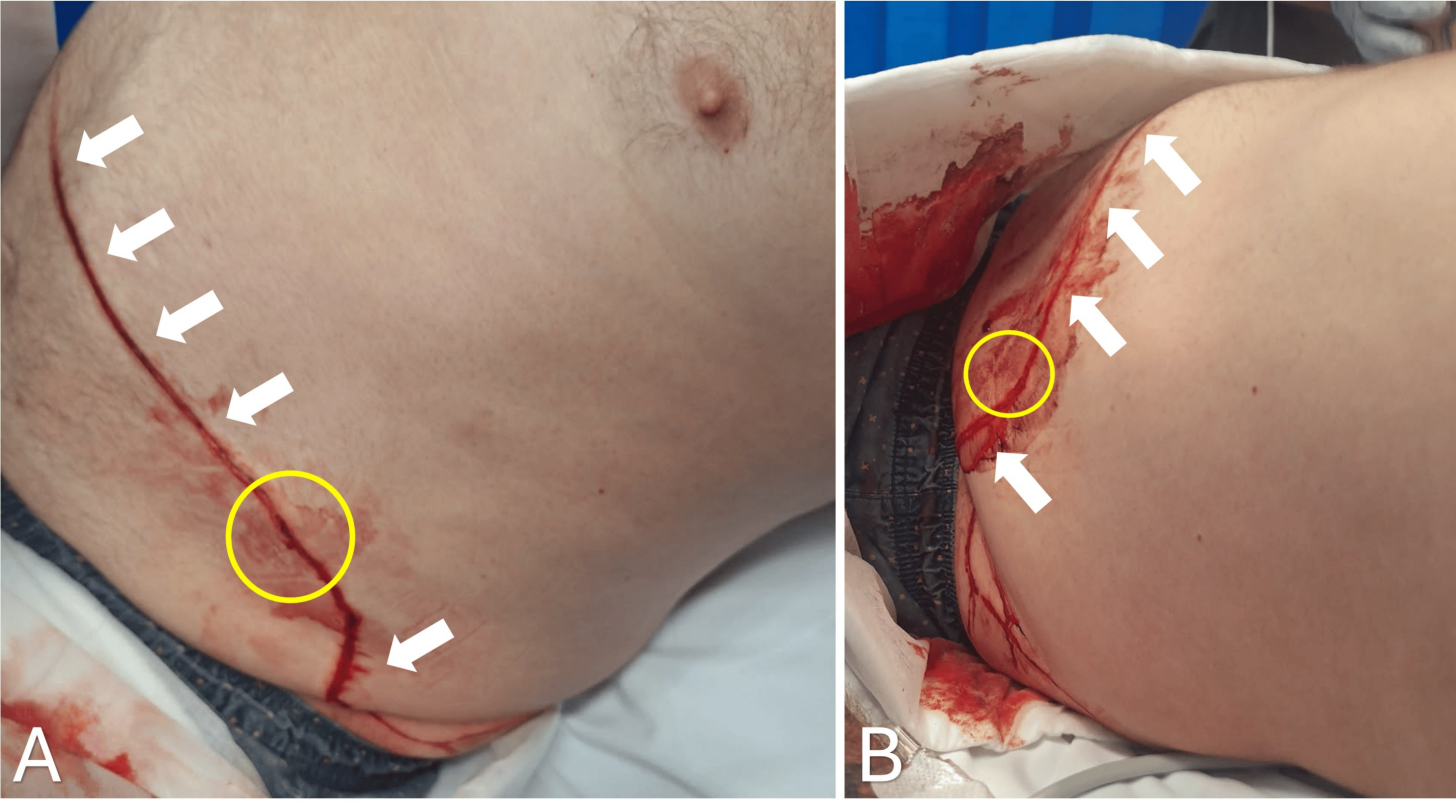
Images demonstrating skin laceration to the right mid-abdomen extending towards the left flank (demonstrated with white arrows) and pinhole skin breach (area circled in yellow) A: anterior view; B: lateral view

A bedside physical examination revealed a superficial skin abrasion extending from the right mid-abdomen to the left flank with a small pinhole skin breach (Figure 1). His abdomen was soft, with focal tenderness along the skin injury.

Initial haematological investigations demonstrated normal haemoglobin (Hb), white cell count (WCC), and lactate levels (Table 1).

**Table 1:** Patient's haematological investigations demonstrating normal Hb, WCC, and lactate levels Hb: haemoglobin; WCC: white cell count

Haematological investigations	Reference range	Patient's results
Hb	110-165 g/L	151 g/L
WCC	4.0-11.0×10^9^/L	10.1×10^9^/L
Lactate	0.5-2.2 mmol/L	0.6 mmol/L

**Figure 2:**
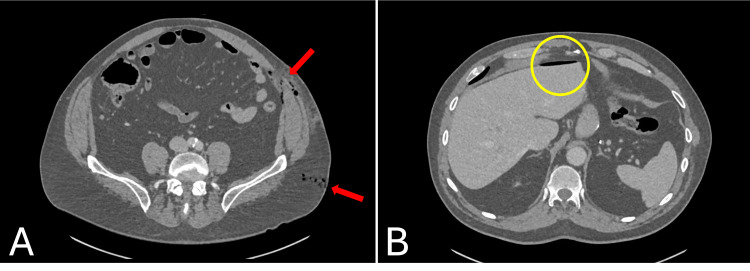
CT scan demonstrating free gas locules in subcutaneous, preperitoneal, and intraperitoneal space (as indicated by red arrows in image A) and free gas above the liver (as indicated by yellow circle in image B) CT: computed tomography

The patient was kept fasted and admitted for observation. A repeat abdominal examination approximately two hours later revealed worsening abdominal pain with guarding and rebound tenderness in the para-umbilical region, consistent with peritonism. A computed tomography (CT) scan was subsequently performed, which demonstrated pneumoperitoneum with gas locules adjacent to the descending colon in the left lower quadrant with no definite bowel wall discontinuity or free fluid identified (Figure 2). In view of evolving clinical deterioration and the CT findings, the decision was made to proceed with diagnostic laparoscopy to investigate the suspected peritoneal breach and intra-abdominal injury.

**Figure 3:**
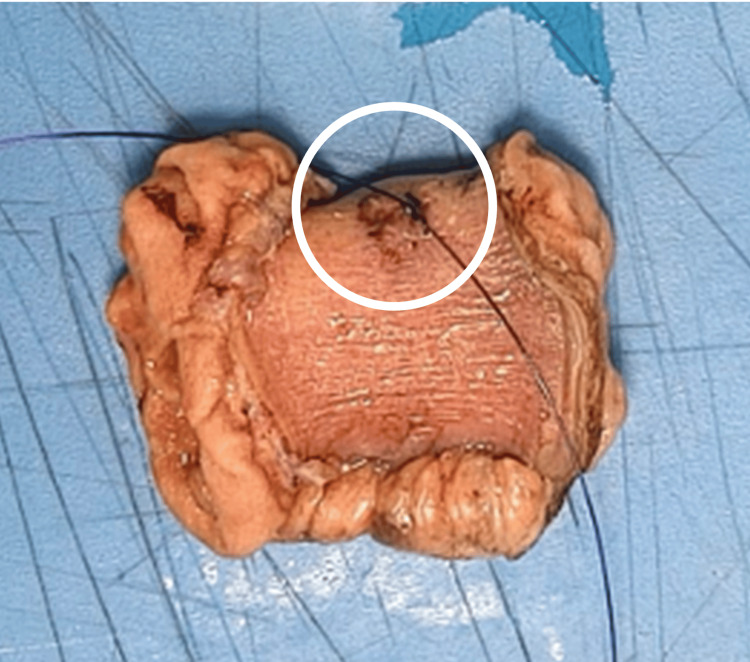
Resected segment of the small bowel with associated serosal bruise marked intraoperatively by suture (as circled in the image)

Two small peritoneal breaches (each <1 cm) were identified on the left side of the abdomen, along with an area of serosal bruising on an adjacent loop of small bowel during diagnostic laparoscopy. Given the evidence of small bowel injury, an intraoperative decision was made to convert to a midline exploratory laparotomy. No additional intra-abdominal injuries were identified. The bruised segment of small bowel was resected, and a primary end-to-end anastomosis was performed (Figure 3).

**Figure 4:**
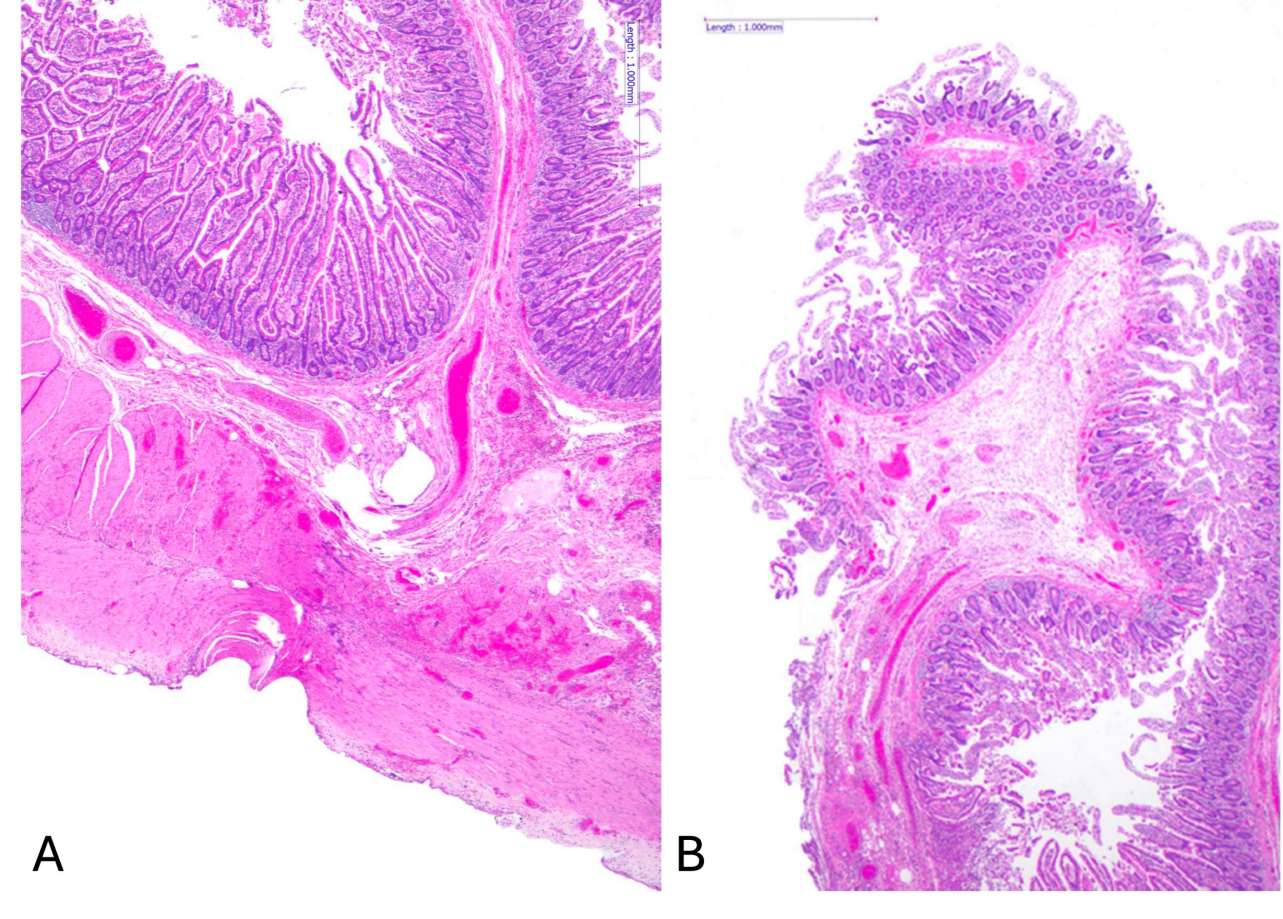
Histopathological examination of small bowel resection with area of interest demonstrating features of focal ischaemic-type changes. A: features of subserosal haemorrhage and inflammation. B: features of mucosal withering Scale bar represents 1,000 mm; magnification 2×, for images A and B

Histopathological examination of the resected small bowel segment demonstrated focal ischaemic-type changes with superficial mucosal necrosis and an associated transmural neutrophilic infiltrate, haemorrhage, and congestion, findings consistent with small bowel contusion (Figure 4). The patient had an uneventful postoperative course and was discharged a week postoperatively.

## Discussion

IHPFII are rare causes of traumatic injury reported in the literature. The non-dominant hand is the most frequently injured site [2]. It is estimated that skin penetration can occur at pressures as low as 100 psi [2,3]; however, most industrial high-pressure injectors operate at pressures of up to 10,000 psi [2,3].

The pathophysiology of IHPFII involves several mechanisms: direct physical injury to the skin, chemical injury from the injected material (which may result in increased oedema and inflammation), and contamination with microorganisms (bacterial, viral, or fungal), potentially leading to infections [1-3].

In the context of intra-abdominal injury, these mechanisms may produce a spectrum of clinical presentations, ranging from simple abdominal wall contusions or haematomas to solid organ injury with haemoperitoneum and/or hollow viscus perforation resulting in peritonitis [1].

The nature of the injected fluid plays a significant role in predicting injury severity. Chemical substances such as paint, gasoline, grease, and oil are associated with more severe inflammation and tissue destruction, often necessitating more aggressive management compared with water-based fluid injuries [2-5]. The reported risk of infection following IHPFII ranges from 20% to 35% in the available literature [2,6], likely due to the water source frequently being contaminated wastewater or river water [6]. In the context of this case, the injected fluid in this instance was just water without known chemicals. 

Given the high velocity and energy transmitted by industrial high-pressure injectors, patients presenting with these injuries should be managed according to the Advanced Trauma Life Support (ATLS) principles [2,7]. However, as demonstrated in our case, the initial physical examination may be falsely reassuring. A high index of suspicion is therefore essential, and serial abdominal examinations should be performed to detect evolving clinical changes. In this case, the patient's worsening abdominal pain prompted further investigation with a CT scan which demonstrated pneumoperitoneum.

Historically, the presence of pneumoperitoneum has been considered indicative of hollow viscus injury and, in the setting of penetrating abdominal trauma, often prompted exploratory laparotomy [8]. However, this approach has evolved in recent years due to the high rate of non-therapeutic laparotomies. Current guidelines advocate selective non-operative management for stable, asymptomatic patients, with immediate laparotomy reserved for those with haemodynamic instability, peritonitis, or impalement [9].

In contrast, the existing literature indicates that CT-detected pneumoperitoneum has a low predictive value for intra-abdominal injury in blunt abdominal trauma. Nevertheless, associated findings, such as free fluid, a seatbelt sign, or radiological evidence of bowel injury, combined with pneumoperitoneum significantly increase the likelihood of clinically relevant injury [10].

Abdominal trauma resulting from an industrial high-pressure injector does not fit neatly into either the penetrating or blunt trauma categories but instead represents a unique mechanism combining high-velocity penetration and hydroblast injury [7]. Consequently, careful clinical judgement and a high index of suspicion are required to guide management. In our case, diagnostic laparoscopy was indicated due to the patient's evolving symptoms and concern for hollow viscus injury in the presence of pneumoperitoneum. Identification of a peritoneal breach and serosal bruising of the small bowel warranted conversion to exploratory laparotomy, allowing adequate exposure to exclude additional intra-abdominal injuries.

## Conclusions

IHPFII to the abdomen are rare but potentially serious causes of abdominal trauma and should be managed with close observation, appropriate antimicrobial therapy, and surgical intervention when indicated. The management of these injuries does not align neatly with traditional classifications of blunt or penetrating abdominal trauma; rather, they represent a unique combination of high-velocity penetrating and hydroblast injury. Consequently, a high index of suspicion and sound clinical judgement are essential to ensure timely diagnosis and to avoid missed intra-abdominal injuries.

## References

[1] Go SJ, Sul YH, Ye JB, Choi JH, Kim JS (2017). Intraperitoneal injury due to a high-pressure water jet. Trauma Mon.

[2] Cejin MC, Koyfman A, Long B (2025). High risk and low incidence diseases: high-pressure injection injury. Am J Emerg Med.

[3] Rodríguez-Villar S, Kennedy RC, Dall'Antonia M, Menichetti CP (2019). Management of industrial high-pressure fluid injection injuries (IHPFII): the Water Jetting Association (WJA) experience with water driven injuries. Eur J Trauma Emerg Surg.

[4] Sharma OP, Oswanski MF (2002). Hydroblast intra-abdominal organ trauma. J Emerg Med.

[5] Estrera AL, Aucar JA, Wall MJ, Granchi TS, Mattox KL (1999). Hydroblast injuries to the small bowel and inferior vena cava. J Trauma Acute Care Surg.

[6] Costello MW, Bolling RP, Gonzalez RP (2008). Intra-abdominal injury as a result of high-pressure water injection. J Trauma Acute Care Surg.

[7] Galvagno SM Jr, Nahmias JT, Young DA (2019). Advanced Trauma Life Support® update 2019: management and applications for adults and special populations. Anesthesiol Clin.

[8] Broder J (2011). Imaging abdominal and flank trauma. Diagnostic Imaging for the Emergency Physician.

[9] Biffl WL, Leppaniemi A (2015). Management guidelines for penetrating abdominal trauma. World J Surg.

[10] Marek AP, Deisler RF, Sutherland JB (2014). CT scan-detected pneumoperitoneum: an unreliable predictor of intra-abdominal injury in blunt trauma. Injury.

